# Bilateral macular hemorrhage as a complication of drug-induced anemia: a case report

**DOI:** 10.1186/1752-1947-3-16

**Published:** 2009-01-13

**Authors:** Rubens N Belfort, Bruno F Fernandes, André Romano, Ricardo Nose, Jonathan Cools-Lartigue, Eduardo V Navajas, Garles MM Vieira, Renato Delascio Lopes, Rubens Belfort

**Affiliations:** 1 Henry C Witelson Ocular Pathology Laboratory, Ophthalmology Department, McGill University, Montreal, QC, Canada; 2 Vision Institute, Federal University of São Paulo, Rua Botucatu, 820 Vila Clementino, São Paulo, Brazil; 3 Faculdade de Medicina da Universidade Metropolitana de Santos-UNIMES, Rua Conselheiro Saraiva, 31 – Vila Nova, Santos, Brazil; 4 Department of Hematology, Servidor Municipal de São Paulo, Rua Castro Alves, 60 – Sao Paulo, SP Brazil; 5 Internal Medicine Department, Federal University of São Paulo, Rua Botucatu, 820 Vila Clementino, São Paulo, SP, Brazil

## Abstract

**Introduction:**

Bilateral macular hemorrhage is a rare ocular finding and to the best of our knowledge, this is the first report of such hemorrhages as a presentation of drug-induced anemia.

**Case presentation:**

We describe the case of a 14-year-old Caucasian boy who presented with a toxoplasmic retinochoroiditis and was treated with sulfadiazine and pyrimethamine. Three months later, he presented with a bilateral macular hemorrhage as a complication of a toxic induced anemia.

**Conclusion:**

Our patient presented with toxic anemia secondary to the treatment of a very common disease, ocular toxoplasmosis. Prophylactic use of folinic acid could prevent such complications but in many cases, it is not prescribed owing to its cost or is mistakenly substituted with folic acid, which does not present as a valid substitute.

## Introduction

Toxoplasmosis is a very common usually benign infection that becomes clinically important in the case of pregnant women, immunocompromised patients or when it affects the eye. Up to 19% of infected patients can present with ocular retinochoroiditis at some point after being infected. The patients with ocular toxoplasmosis are usually treated with an association of sulfadiazine/pyrimethamine or trimethoprim/sulfamethoxazole for 4 to 6 weeks [1,2].

## Case presentation

A 14-year-old boy presented to the emergency room with a 1-week history of sore throat, fever and malaise. He had been diagnosed with ocular toxoplasmosis 3 months before presentation and treated with sulfadiazine (4 g/day), pyrimethamine (25 mg/day) and folinic acid (7.5 mg/day). To note, even though folinic acid was prescribed, the patient was taking folic acid instead because the latter is much less expensive. He had been using the medication for two and a half months, until 15 days before coming to the emergency room. Personal and familial past medical history were unremarkable. As the blood work revealed anemia and leucopenia (Hb: 4.2 gm/dL; 2100 leukocytes/μL [300 neutrophils]; 348,000 platelets/mL), the boy was admitted for further hematologic investigation.

The patient presented with an intense anemia and severe neutropenia. Bone marrow aspirate was normocellular with hyperplasia of the erythrocytic lineage, with megaloblastic and dysplasic elements. The granulocytic lineage showed hypocellularity and dysgranulopoiesis with maturation asynchronism. The megakaryocytic lineage was preserved and there was a low-grade plasmacytosis of 6.2% of the total cells. These findings were compatible with drug-induced bone marrow toxicity in response to pyrimethamine or sulfur.

Four days after admission, the patient complained of poor vision in the left eye. Visual acuity was 20/30 in the right eye and counting fingers (CF) at 1 meter in the left. Pupillary reaction, ocular motility, intra-ocular pressure (12 mmHg AO), and anterior segment were normal. Fundus exam of the right eye revealed sub-retinal hemorrhages, intra-retinal hemorrhages with a white center and a retinochoroidal scar inferior to the optic nerve head. This retinal scar was compatible with the toxoplasmic infection 3 months before. Fundus exam of the left eye showed a macular hemorrhage. It was decided to observe and elevated decubitus was recommended so that the blood could sediment and clear the visual axis.

**Figure 1:**
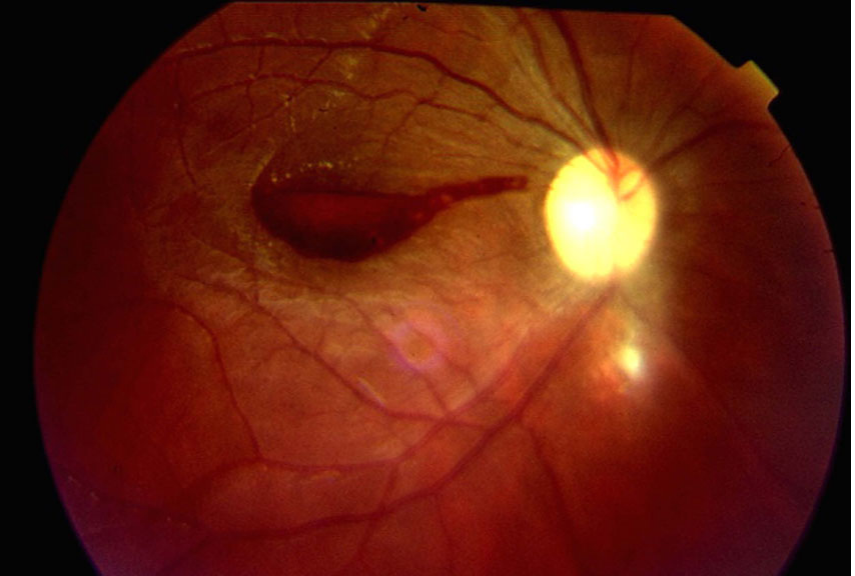
**Retinography of the right fundus shows a preretinal hemorrhage at the macula and a retinochoroiditis scar inferior to the optic disk**.

**Figure 2:**
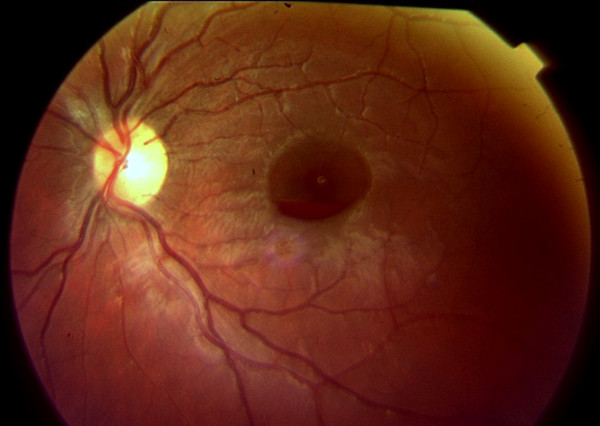
**Retinography of the left fundus shows a preretinal hemorrhage in absorption**.

Two days later, his visual acuity dropped to CF at 1 m in the right eye, without anterior segment abnormalities. Visual acuity in the left eye had improved to 20/60 at that time. Fundus exam revealed bilateral macular hemorrhages and some small preretinal hemorrhages in the mid-periphery of the right eye (Figures 1 and 2). At that point, the patient's blood work revealed an improvement in the anemia and leucopenia (Hb: 10.8 gm/dL; 3600 leucocytes/μL; 268,000 platelets/mL).

Again elevated decubitus was recommended. After 4 days, the visual acuity was 20/200 in the right eye and 20/30 in the left eye, with improvement in the retinal hemorrhages and inferior deposition of the preretinal blood in both eyes, clearing the visual axis. After 4 weeks of follow-up, visual acuity was 20/20 in both eyes.

## Discussion

The rare, classically called "sub-hyaloid" or "preretinal" macular hemorrhage is actually located between the neuro fiber layer and the inner limiting membrane.

Causes of this hemorrhage include Valsalva maneuver, diabetic retinopathy, retinal branch vein occlusion, ruptured macro-aneurism of the retina and *shaken baby *syndrome. Other rare causes include thrombocytopenia secondary to bone marrow aplasia, leukemia, auto-immune hemolytic anemia, aplastic anemia secondary to drug toxicity, severe head trauma, bleeding secondary to intercourse and intraocular pressure variation during refractive surgery (LASIK). Bilateral macular hemorrhages are even more uncommon, with a few reports associated with cases of head trauma, hemolytic anemia and thrombocytopenia [3].

In our report, the patient presented the hemorrhage while admitted in the hospital and being constantly evaluated. Causes for Valsalva retinopathy were excluded and anemia was the most likely etiology, given that he did not present with thrombocytopenia during his time at the hospital. The explanation for retinal hemorrhages in the presence of anemia without thrombocytopenia is not clear. Factors such as anoxia, venous stasis, angiospasm, increased capillary permeability, and thrombocytopenia have been implicated in the pathogenesis of anemic retinopathy [4].

The hematologic investigation, including bone marrow aspirate, was compatible with drug-induced bone marrow toxicity in response to pyrimethamine or sulfur [5-8].

Both drugs interfere with the conversion of folate to its active form, tetrahydrofolate, essential for DNA synthesis. The inadequate DNA replication leads to deficient hematopoietic cell division, with megaloblastic features, and granulocytic alterations described above. Clinically, patients present with anemia which may be accompanied by leucopenia and thrombocytopenia.

Thirty days following the cessation of the medication, the patient improved, supporting our hypothesis of drug-induced medullary toxicity.

## Conclusion

Bilateral macular hemorrhages secondary to anemia are rare and to the best of our knowledge, this is the first report of subhyaloid hemorrhage secondary to drug-induced bone marrow toxicity.

The use of folinic acid in the treatment of toxoplasmosis could prevent complications from the use of dihydrofolate reductase inhibitors such as pyrimethamine but is expensive and not always provided by public health systems in different countries. The use of folic acid should not be considered as an alternative because it does not prevent drug-induced toxicity. Physicians should be aware of this rare but serious complication of ocular toxoplasmosis treatment.

## Abbreviations

OU: both eyes

## Competing interests

The authors declare that they have no competing interests.

## Authors' contributions

RNB, BFF, EN and AR were the ophthalmologists taking care of the patient, GMMV and RDL were the hematologists treating the patient. JCL, RN and RB Jr were major contributors in writing the manuscript. All authors read and approved the final manuscript.

## Consent

Written informed consent was obtained from the patient for publication of this case report and any accompanying images. A copy of the written consent is available for review by the Editor-in-Chief of this journal.
